# The Future of Pharmacogenomics: Integrating Epigenetics, Nutrigenomics, and Beyond

**DOI:** 10.3390/jpm14121121

**Published:** 2024-11-27

**Authors:** Jeffrey A. Shaman

**Affiliations:** Coriell Life Sciences, Philadelphia, PA 19112, USA; jshaman@coriell.com

**Keywords:** pharmacogenomics, epigenetics, nutrigenomics, microbiome, protein interactions, exosomes, metabolomics, personalized medicine

## Abstract

Pharmacogenomics (PGx) has revolutionized personalized medicine by empowering the tailoring of drug treatments based on individual genetic profiles. However, the complexity of drug response mechanisms necessitates the integration of additional biological and environmental factors. This article explores integrating epigenetics, nutrigenomics, microbiomes, protein interactions, exosomes, and metabolomics with PGx to enhance personalized medicine. In addition to discussing these scientific advancements, we examine the regulatory and ethical challenges of translating multi-omics into clinical practice, including considerations of data privacy, regulatory oversight, and equitable access. By framing these factors within the context of Medication Adherence, Medication Appropriateness, and Medication Adverse Events (MA^3^), we aim to refine therapeutic strategies, improve drug efficacy, and minimize adverse effects, with the goal of improving personalized medicine. This approach has the potential to benefit patients, healthcare providers, payers, and the healthcare system as a whole by enabling more precise and effective treatments.

## 1. Introduction

Pharmacogenomics (PGx) represents a cornerstone of personalized medicine, focusing on how individual genetic differences influence drug response and efficacy. PGx combines genetic testing with clinical pharmacology to identify how a patient’s genetic makeup can affect their response to specific medications. It focuses particularly on genes that encode drug-metabolizing enzymes, such as CYP450 enzymes, and drug transporters, like P-glycoprotein. These proteins influence how medications are absorbed, distributed, metabolized, and eliminated by the body, which can affect both drug efficacy and the risk of adverse effects. For example, variations in the CYP2C19 gene can alter the metabolism of clopidogrel, impacting its effectiveness as an antiplatelet medication. By tailoring medical treatments based on genetic information, PGx supports the optimization of therapeutic outcomes and minimization of adverse effects. Despite significant advances, the full potential of personalized medicine remains to be realized, in part due to the complex interplay of various genetic, biological, and environmental factors that influence drug response.

**Figure 1:**
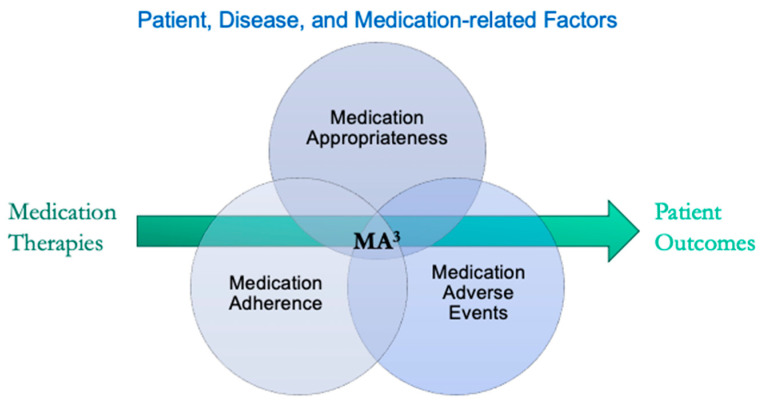
The pharmacotherapy process from prescribing to patient outcomes. Outcomes are shaped by various patient, disease, and medication-related factors, which are categorized under medication adherence, appropriateness, and adverse events (MA^3^).

Personalized medicine aims to optimize therapeutic outcomes by addressing individual variability in drug response. The concept of MA^3^—Medication Adherence, Medication Appropriateness, and Medication Adverse Events—provides a structured framework for understanding how these factors influence therapeutic outcomes (Figure 1). By addressing the influencing factors of MA^3^, healthcare providers can better tailor medication regimens to individual patients, thereby enhancing the overall effectiveness of personalized medicine. 

Medication Adherence refers to how well patients follow their prescribed medication regimens, which is crucial for achieving desired health outcomes. Complex drug regimens, high pill burdens, concern about side effects, low perceived need or efficacy, cost, and limited patient engagement in treatment decisions can lead to poor adherence. Practical issues like forgetfulness, complex drug regimens, impaired cognition, misunderstood medication instructions, low health literacy, and lack of social support further contribute to poor adherence.

Medication Appropriateness involves selecting the right medication for the right patient, considering their unique genetic, biological, and environmental factors. Inappropriate medication choices, such as insufficient dosing in oncology treatments, selecting drugs prone to significant interactions, or prescribing a duplicative or unjustified medication therapy can lead to ineffective treatment and unintended outcomes. Risks of drug–drug, drug–disease, and drug–gene interactions, as well as impractical directions, can also complicate treatment.

Medication Adverse Events arise from dose-related issues, idiosyncratic reactions, long-term exposure, withdrawal, allergic reactions, and pharmacodependence. External factors like drug interactions or disease can also alter an individual’s drug metabolism, a phenomenon known as phenoconversion, further increasing the risk of therapy failure.

To further enhance the personalization of medication regimens, integrating additional layers of biological information—such as genetic, epigenetic, and metabolic data—within the MA^3^ framework offers a comprehensive approach to optimizing therapeutic outcomes. Recent advancements and standard pharmacy practices highlight the need to integrate additional layers of biological information to refine rational prescribing practices. This article explores the integration of epigenetics, nutrigenomics and nutrition, the microbiome, protein interactions, exosomes, and metabolomics with PGx within the context of MA^3^, providing a comprehensive view of future directions in personalized medicine. While these approaches hold great promise, they are not yet widely available or integrated into routine clinical practice due to significant cost barriers, the technical challenges of high-throughput data generation and analysis, and a lack of robust clinical outcome data to support their widespread implementation. By incorporating these omics layers alongside strategies to overcome the challenges, healthcare providers and researchers can aim to enhance the precision, safety, and effectiveness of personalized treatments, moving beyond genetics to include dynamic and modifiable influences on drug outcomes.

## 2. Epigenetics

Epigenetics refers to modifications in gene expression that do not entail altering the DNA sequence. These pre- and post-translational modifications, including DNA methylation, histone modification, and non-coding RNA interactions [1] can modulate gene expression, influenced by environmental factors, lifestyle, toxins, stress, and disease states. Some of these changes may persist for generations.

### 2.1. Epigenetic Influence on Drug Response

An example of epigenetic influence on drug response is the modification of the *CYP2E1* gene expression, which plays a crucial role in acetaminophen metabolism and toxicity. The decrease in DNA methylation in the promotor and other sites of the *CYP2E1* gene increase its expression, leading to increased metabolism and production of the toxic metabolite N-acetyl-p-benzoquinone imine (NAPQI), thereby increasing the risk of acetaminophen toxicity [2]. Additionally, microRNA miR-378 upregulation has been shown to be associated with the translational repression of *CYP2E1* [3], and histone acetylation changes may be associated with *CYP2E1* gene expressions [4] and rates of *CYP2E1*-mediated drug metabolism [5]. Similarly, methylation and histone modifications affecting the *ABCB1* gene, which encodes the multiple drug resistance protein P-glycoprotein drug transporter, have been linked to variations in chemotherapy response, with significant implications for treatment efficacy and toxicity [6,7,8]. Of particular interest for future therapeutic strategies, FDA-approved epigenetic inhibitors, including histone deacetylase inhibitors, histone acetyltransferase inhibitors, histone methyltransferase inhibitors, and DNA methyltransferase inhibitors, have shown promise in modulating gene expression changes and may provide new therapeutic options [9].

### 2.2. Assaying Epigenetic Changes

Epigenetic modifications can be assayed using various techniques [10]:

- DNA Methylation: Bisulfite sequencing converts unmethylated cytosines to uracil, allowing for the identification of methylated regions through sequencing. Methylation-specific PCR and microarray-based methods can also be used.
- Histone Modifications: Chromatin immunoprecipitation (ChIP) followed by sequencing (ChIP-seq) can identify histone modifications associated with specific genomic regions. Mass spectrometry can also be used for a detailed analysis of histone modifications.
- Non-coding RNAs: RNA sequencing (RNA-seq) can quantify and characterize non-coding RNAs, including microRNAs (miRNAs) and long non-coding RNAs (lncRNAs) [11].

### 2.3. Integrating Epigenetics into Personalized Medicine

The integration of epigenetic data with traditional pharmacogenomics offers a profound opportunity to deepen our understanding of individual drug responses. By combining genetic predispositions with epigenetic markers, we can more accurately identify patients at a heightened risk of adverse drug events or those who are likely to derive significant benefit from specific therapies. 

## 3. Nutrigenomics and Nutritional Interactions

Nutrigenomics examines how diet and genetics interact to influence health and disease. It explores how specific nutrients or dietary patterns can modify gene expression, affect metabolism (pharmacokinetic and pharmacodynamic), and interact with endogenous enzymes, consequently impacting health outcomes. Understanding a patient’s genetic and non-genetic responses to nutrients can positively influence various health aspects, including weight loss, disease prevention, athletic performance, high blood pressure, cholesterol levels, reactions to caffeine, and drug response. 

### 3.1. Nutritional Influence on Health and Drug Response

A well-characterized example of nutrigenomics is the influence of dietary folate and riboflavin on gene expression. Folate is essential for the one-carbon metabolism pathway, which is crucial for DNA synthesis and methylation. Adequate folate intake can regulate gene expression by influencing DNA methylation patterns, which in turn can impact various health outcomes, including cancer risk, cardiovascular disease, diabetes, and fetal neural development. Individuals with the C677T polymorphism in the methylenetetrahydrofolate reductase (*MTHFR*) gene may require different levels of folate to maintain proper DNA methylation and prevent diseases associated with folate deficiency [12].

In addition to nutrigenomics, drug–nutrient–genome interactions (DNGIs) play a critical role in modulating drug efficacy and safety. One extensively studied interaction is between grapefruit juice and various medications. Grapefruit juice inhibits the metabolizing enzyme CYP3A4, leading to increased plasma concentrations of drugs metabolized by it, such as calcium channel blockers, statins, immunosuppressants, anti-infective agents, and others, potentially causing adverse effects [13,14,15]. Other dietary supplements such as cranberry products, St John’s wort, and ginkgo biloba are also known to impact CYP2C9, CYP3A4, CYP2C19, CYP2E1 and other drug-metabolizing enzymes, altering medication effectiveness and safety [16]. Recent studies highlight how specific foods can influence DNA methylation patterns, with implications for personalized nutrition. For instance, cream and spirits have been associated with methylation changes in genes like *CLN3*, *PROM1*, *DLEU7*, *TLL2*, and *UGT1A10* [17]. Additionally, green tea, rich in epigallocatechin gallate (EGCG), and cruciferous vegetables containing sulforaphane have been shown to modify DNA methylation patterns [18], which may affect gene expression, influencing drug metabolism and therapeutic outcomes. Other DNGIs examples include flavonoids found in fruits and vegetables, compounds like quercetin and licorice root, and vitamin K-rich foods [16,19].

Understanding these intricate interactions underscores the importance of personalized nutrition. It plays a vital role in optimizing therapeutic outcomes and minimizing adverse effects.

### 3.2. Assaying Nutrigenomic Interactions

Nutrigenomic interactions can be assessed [20] through the following:

- Dietary Surveys and Biomarkers: Dietary intake can be assessed using food frequency questionnaires (FFQs), 24 h dietary recalls, and food diaries. Biomarkers such as blood levels of vitamins and minerals can provide objective measures of nutrient availability.
- Genotyping and Sequencing: Genotyping and sequencing platforms can identify genetic variants that interact with dietary factors.
- Gene Expression Analysis: RNA sequencing (RNA-seq) and quantitative PCR (qPCR) can be used to study changes in gene expression in response to dietary interventions.

### 3.3. Integrating Nutrigenomics into Personalized Medicine

Adding nutrigenomics and drug-nutrient-genome interactions (DNGIs) into rational prescribing offers the potential to align dietary recommendations with drug prescriptions. Personalized nutrition plans can optimize drug therapy, reducing adverse effects and enhancing efficacy. This integration can improve medication adherence and appropriateness and reduce adverse events (MA^3^), thus improving therapeutic outcomes. 

## 4. Microbiome

The microbiome consists of trillions of microorganisms living in and on our bodies, especially in the gut. These microorganisms play a crucial role in drug metabolism and response by modulating the bioavailability of drugs. 

### 4.1. Microbiome Influence on Drug Response

The influence of gut microbiota on irinotecan metabolism is a well-documented example. The bacterial enzyme β-glucuronidase can convert the inactive form of irinotecan back to its active form (i.e., SN-38) in the gut, leading to toxicity [21]. Another example is the impact of the microbiome on digoxin metabolism, where certain gut bacteria, such as *Eggerthella lenta*, can inactivate digoxin, affecting its efficacy in treating heart failure and atrial fibrillation [22,23,24]. Interestingly, sulfasalazine (salicylazosulfapyridine), a drug used to treat inflammatory bowel disease, is metabolized by gut bacteria into its active form, 5-aminosalicylic acid (5-ASA) [25]. This conversion is essential for the drug’s therapeutic effect, demonstrating that the microbiome can also directly activate prodrugs to their effective forms. 

### 4.2. Assaying Microbiome Changes

Microbiome analysis [26] can be performed using the following:

- 16S rRNA Sequencing: This technique sequences the 16S ribosomal RNA gene, which is highly conserved among bacteria, allowing for the identification and quantification of bacterial species in a sample.
- Metagenomics: Whole-genome shotgun sequencing can provide a comprehensive view of the microbial community by sequencing all the genetic material in a sample.
- Metatranscriptomics and Metaproteomics: These approaches study the active gene expression (RNA) and protein production of the microbiome, providing insights into the functional activity of microbial communities.

### 4.3. Integrating Microbiome Data into Personalized Medicine

Clearly, we are beginning to appreciate the impact that gut bacteria have on medication safety and efficacy. And although not yet routinely integrated into clinical testing, incorporating microbiome data with other patient information can offer a more comprehensive understanding of drug metabolism and efficacy, potentially leading to improved therapeutic outcomes. 

## 5. Protein Interactions

Proteomics refers to the large-scale study of proteins, including their structures, functions, and interactions. Unlike the static nature of the genome, the proteome reflects the dynamic state of a patient’s health, providing real-time insights into physiological and pathological processes. Proteomics is inextricably linked to genetics, but often provides a more immediate snapshot of the current state of health and potential future disease development. Protein interactions, including those involving drug-metabolizing enzymes, transporters, and plasma proteins, are intrinsic to the body and significantly impact drug response. 

### 5.1. Protein Influence on Drug Response

Post-translational modifications such as ubiquitination, phosphorylation, and glycosylation can alter the function, activity, and stability of proteins. For instance, the ubiquitination and deubiquitination of the ABCB1 (P-glycoprotein) protein can influence its degradation, stability, and function. ABCB1 is a crucial transporter protein that plays a significant role in the efflux of drugs across cell membranes. Ubiquitination can target ABCB1 for proteasomal degradation, which alters cellular drug concentrations and influences drug resistance and efficacy. This process is particularly relevant in cancers such as breast and colorectal tumors, where the overexpression or dysregulation of ABCB1 has been associated with chemotherapy resistance [27]. In contrast, ubiquitination may enhance the efficacy of chemotherapeutic agents like paclitaxel and doxorubicin by targeting ABCB1 for degradation, potentially improving tumor prognosis.

Additionally, proteomics can identify biomarkers with the potential to predict drug response in much the same way as genetics can. The presence of specific protein isoforms or expression levels can serve as direct, point-in-time biomarkers for predicting the efficacy and toxicity of certain drugs. This understanding can help in the tailoring of drug therapies to individual patients based on their proteomic profiles and can even be specific to the site of action, drug transport, or biofluid.

Moreover, the binding of drugs to plasma proteins such as albumin affects the drugs’ distribution, free concentration, and elimination. Proteomic studies can quantify the extent of protein-drug binding and identify conditions that alter this interaction. For example, diseases that affect protein levels, such as liver disease, can alter drug binding, necessitating dosage adjustments.

### 5.2. Assaying Proteomics

Protein interactions can be studied [28] using the following:

- Mass Spectrometry: This technique can identify and quantify proteins and their post-translational modifications, providing insights into protein function and interactions.
- Co-Immunoprecipitation (Co-IP): This method isolates protein complexes from cell lysates using specific antibodies, allowing for the identification of interacting proteins.
- Yeast Two-Hybrid Screening: This genetic technique can detect protein–protein interactions by linking the interaction of two proteins to the activation of a reporter gene.

### 5.3. Integrating Protein Interaction Data into Personalized Medicine

Incorporating proteomic data into personalized medicine can refine genetic predictions of drug response and safety. While genetics is a great proxy for protein expression, assays that can identify protein structure and function—directly and in real-time—will be a boon to enhancing therapeutic outcomes. 

## 6. Exosomes

Exosomes are small extracellular vesicles that facilitate intercellular communication by transferring proteins, lipids, and nucleic acids between cells. They play a significant role in various physiological and pathological processes and can be assayed from blood, urine, cerebrospinal fluid, breast milk, ascitic fluid, and other biological fluids. 

### 6.1. Exosome Influence on Drug Response

Exosomes can influence drug metabolism and efficacy by transporting drug-metabolizing enzymes, regulatory molecules, and medications between cells. For example, exosomes derived from drug-resistant cancer cells can transfer resistance traits to sensitive cells, impacting chemotherapy outcomes [29]. This transfer of resistance traits can include proteins, lipids, and nucleic acids that contribute to drug resistance mechanisms [30]. 

Additionally, exosomes have been shown to contain CYP mRNAs and active enzymes, suggesting that the metabolism of medications, alcohol, and tobacco may not be limited to the liver and may be transported to sites where needed or could even be utilized within the biological fluid and other extracellular environments [31]. 

Exosomes also show promise as biomarkers for drug response and disease state monitoring. Their content reflects the physiological and pathological conditions of their cells of origin, making them valuable for non-invasive diagnostics and personalized medicine strategies [32]. A notable example is their potential use as biomarkers for transplant rejection. Recent studies have demonstrated that exosomes carry immune-related molecules such as donor-specific antigens and inflammatory cytokines, which may be detected in urine, to monitor graft health and predict rejection events, providing a valuable tool for post-transplant care [33]. 

### 6.2. Assaying Exosomes

Exosomes can be isolated and analyzed [34] using the following:

- Ultracentrifugation: This method separates exosomes based on their size and density using high-speed centrifugation.
- Size Exclusion Chromatography (SEC): This technique separates exosomes from other extracellular vesicles and proteins based on their size.
- Affinity Selection: This method can characterize and quantify exosomes based on their surface markers.
- Exosomal RNA Sequencing: This approach sequences RNA contained within exosomes to identify potential biomarkers for drug response.

### 6.3. Integrating Exosome Data into Personalized Medicine

Incorporating exosome profiling into PGx can provide insights into dynamic changes in drug response mechanisms. Exosome-derived biomarkers can help predict drug efficacy and resistance, offering a novel approach to personalized medicine. This integration holds potential to enhance medication appropriateness, adherence, and reduce adverse events by providing real-time, non-invasive insights into traditionally difficult-to-assay biological environments, offering a novel approach to personalized medicine. 

## 7. Metabolomics

Metabolomics is the comprehensive study of metabolites—small molecules produced during metabolic processes within biological systems—within cells, tissues, or organisms. These metabolites represent the products of cellular processes and provide a snapshot of the physiological state of an organism. By examining these metabolites within a biological system, metabolomics offers insights into how the metabolic state changes in response to various factors, including drug treatments, disease state, and environmental factors. 

### 7.1. Metabolomic Influence on Drug Response

Metabolomic profiling can identify biomarkers that could predict how a patient will respond to specific drugs by analyzing metabolic signatures [35,36]. For example, alterations in metabolic pathways such as oxidative phosphorylation, redox metabolism, mitochondrial energy metabolism, fatty acid synthase, or glutaminolysis can confer resistance to chemotherapy agents like bortezomib, carfilzomib, cisplatin, and paclitaxel, highlighting the role of metabolism in drug resistance [37]. Pharmacometabolomics can also help predict pharmacokinetic (PK) properties and pharmacodynamic (PD) effects by analyzing how drug metabolism interacts with the patient’s metabolic state. For instance, metabolites involved in the tricarboxylic acid (TCA) cycle, amino acid metabolism, and fatty acid oxidation can influence the effectiveness and toxicity of anticancer drugs. Consequently, metabolic profiling in early clinical studies can inform whether a drug reaches its target, engages the target, and modulates it in the desired manner [37,38].

Additionally, pharmacometabolomics can help explain the variability in drug response among individuals. Factors such as age, disease state, and co-administered medications can alter metabolic pathways, influencing drug metabolism and response. For example, patients with organ failure and sepsis may exhibit different metabolic profiles [39], making it challenging to determine the appropriate dosing for medications such antibiotics, anticoagulants, and antipsychotics using standard methods [40]. This variability necessitates personalized drug dosing and careful monitoring to optimize therapeutic outcomes.

Furthermore, metabolomics can help identify off-target effects and adverse drug events. By analyzing changes in the metabolome, researchers can detect early signs of drug-induced liver injury, cardiotoxicity, and other adverse effects, leading to improved drug safety.

### 7.2. Assaying Metabolomic Changes

Metabolomic analysis [41] can be performed using the following:

- Mass Spectrometry (MS): This technique identifies and quantifies metabolites based on their mass-to-charge ratio, providing detailed information about metabolic pathways.
- Nuclear Magnetic Resonance (NMR) Spectroscopy: NMR measures the magnetic properties of atomic nuclei, offering a non-destructive way to analyze the chemical composition of metabolites.
- Chromatography: Techniques such as gas chromatography (GC) and liquid chromatography (LC) can separate complex mixtures of metabolites for further analysis by MS or NMR.

### 7.3. Integrating Metabolomics into Personalized Medicine

Integrating metabolomic data with other omics factors can enhance our understanding of how genetic variations influence metabolic pathways and drug responses. This integrative approach can help identify metabolic signatures associated with specific genetic profiles, enabling more precise predictions of drug efficacy and safety. 

## 8. Discussion

The integration of epigenetics, nutrigenomics, microbiomes, protein interactions, exosomes, and metabolomics with pharmacogenomics (PGx) holds tremendous potential for advancing personalized medicine. However, the practical implementation of these integrative approaches presents several challenges, particularly in the context of clinical testing.

### 8.1. Challenges and Opportunities in Testing and Integrating Omics Data

Testing for each biological interaction presents unique challenges, especially in the context of integrating multi-omics data. Assaying epigenetic changes such as DNA methylation and histone modifications involves sophisticated techniques like bisulfite sequencing and ChIP-seq, which are time-consuming and costly. Additionally, the dynamic nature of epigenetic modifications requires longitudinal studies to capture changes over time, further increasing complexity and expense. Advances in single-cell epigenomics and high-throughput sequencing technologies could make epigenetic testing more feasible in clinical settings.

Following epigenetics, nutrigenomics faces challenges of its own. Nutrigenomic studies often rely on subjective dietary assessments—and accurate ingredient information—which can be prone to recall bias. The complex interaction between diet and genetics is influenced by numerous variables, making it difficult to establish clear causal relationships. However, emerging dietary tracking technologies, such as wearable sensors and digital food diaries, could provide more accurate and objective data. 

In parallel, microbiome analysis presents challenges due to the variability of microbiota across different body sites and their sensitivity to environmental factors. Analyzing the microbiome involves techniques like 16S rRNA sequencing and metagenomics, which are complex and resource intensive. The microbiome’s variability across different body sites and its sensitivity to environmental factors add layers of complexity to its study. However, despite these challenges, advances in microbiome analysis, including more rapid and cost-effective sequencing methods, could make microbiome profiling more accessible. Standardizing protocols for microbiome sampling, analysis, and acquiring baseline values will be crucial for its integration into clinical practice.

Similar issues arise when studying protein interactions, which involve methods like mass spectrometry, co-immunoprecipitation, and yeast two-hybrid screening. These techniques require specialized equipment and expertise, limiting their widespread use. Furthermore, these methods are often limited by the need for large sample sizes and by the complexity of protein networks. Fortunately, advances in emerging technologies like proximity ligation assays and advanced mass spectrometry techniques may help reduce some of these barriers. Integration with bioinformatics tools will also be necessary to enhance the interpretation of complex protein networks.

The study of exosomes also presents technical challenges, particularly in the isolation and characterization of these vesicles. Techniques like ultracentrifugation, size exclusion chromatography, and nano-flow cytometry are labor-intensive and resource-demanding. Additionally, the heterogeneity of exosome populations complicates both their study and clinical application. Despite these obstacles, innovations in exosome isolation and analysis—such as microfluidic devices and high-throughput screening methods—could help facilitate their clinical use. Developing standardized protocols for exosome characterization will be essential for their integration into personalized medicine.

Finally, metabolomics, the study of metabolites—small molecules produced during metabolic processes within biological systems—requires advanced technologies like mass spectrometry and nuclear magnetic resonance (NMR) spectroscopy. These technologies are not only expensive but also require significant expertise to operate. Additionally, the metabolome’s sensitivity to environmental factors, diet, and the microbiome introduces further complexity, making its analysis more challenging. However, as analytical tools continue to improve, metabolomics is expected to become increasingly important in predicting drug responses within clinical settings, contributing to more personalized and effective treatment strategies.

### 8.2. Impact of Multi-Omics Integration on Personalized Treatment

Integrating diverse fields such as epigenetics, nutrigenomics, microbiome studies, protein interactions, exosomes, and metabolomics with pharmacogenomics has the potential to revolutionize personalized medicine. By leveraging these multiple layers of biological and environmental data, healthcare providers can gain a deeper understanding of how these factors contribute to drug responses, allowing for more precise and effective treatment strategies.

By incorporating genetic information with epigenetic, dietary, microbiome, and protein interaction data, healthcare providers can achieve a more comprehensive understanding of individual drug responses. This integration not only aids in predicting adverse drug events but also in optimizing dosing strategies, leading to more precise and safer medication regimens. Innovations in these fields promise to enhance the safety and efficacy of medications, thus improving health outcomes for patients.

Additionally, the inclusion of these layers of biological information facilitates the creation of highly personalized treatment plans. These plans take into account a patient’s genetic makeup, lifestyle, and environmental factors, ensuring that treatments are finely tuned to meet individual needs. As a result, personalized treatment strategies can lead to improved patient satisfaction and better overall health outcomes.

Understanding the complex interplay between genes, environmental factors, and disease is crucial for the effective management of chronic conditions. For instance, integrating microbiome data with pharmacogenomics can enhance the treatment of gastrointestinal disorders, while insights from nutrigenomics can inform strategies for managing metabolic diseases. Such a comprehensive view allows for more effective and targeted interventions.

Moreover, integrating omics profiling into pharmacogenomics offers new insights into dynamic changes in drug response mechanisms. Metabolomic and exosome-derived biomarkers, for example, can help predict drug efficacy and resistance, providing opportunities for developing targeted therapies and personalized interventions. As technology advances, these fields will likely reveal even more therapeutic possibilities.

Although the potential of these integrative approaches is immense, many of the assays required to gather these data are currently better suited for research than for routine clinical testing. The complexity, cost, and technical expertise required to perform these assays pose significant barriers to their widespread clinical application. However, as technological advancements continue to improve the precision and efficiency of these assays, we can expect to see greater integration of multi-omics data into personalized medicine. As these assays become more accessible and clinically validated, they will likely transform healthcare by offering clinicians more comprehensive tools to tailor treatments and improve patient outcomes.

### 8.3. MA^3^ as a Key Pillar in Personalized Medicine

The structured concept of MA^3^—Medication Adherence, Medication Appropriateness, and Medication Adverse Events—forms a central framework for integrating multi-omics data into clinical practice. By focusing on these factors, healthcare providers can more effectively address the dynamic and modifiable influences on drug outcomes. This approach ensures that personalized medicine extends beyond genetic profiles to encompass broader patient-specific factors, ultimately optimizing therapeutic outcomes.

### 8.4. Challenges in the Clinical Integration of Multi-Omics: Regulatory and Ethical Considerations

While the integration of multi-omics data with pharmacogenomics holds significant promise for advancing personalized medicine, there are several regulatory and ethical challenges that must be navigated before these approaches can be widely adopted in clinical practice. One of the primary hurdles lies in regulatory oversight and validation. The lack of standardized protocols and regulatory frameworks for multi-omics testing is a critical barrier to its clinical integration. Each “omics” layer involves complex and evolving methodologies that often lack consensus around quality control, data validation, and reproducibility. For instance, while pharmacogenomics testing has been increasingly standardized through agencies like the US FDA, CLIA, and CAP, the integration of data from the microbiome or epigenetic assays has yet to achieve the same level of regulatory clarity. The absence of well-defined regulatory pathways for validating and approving multi-omics diagnostic tests could slow their adoption in clinical workflows.

Additionally, there is a pressing need for regulatory agencies to determine how to classify and approve multi-omics tests—whether as companion diagnostics, lab-developed tests (LDTs), or as part of novel treatment paradigms. In the absence of clear guidelines, developing and commercializing multi-omics tools may be delayed, restricting access for patients who stand to benefit from these advancements

The integration of multi-omics data also raises significant privacy concerns due to the vast amount of highly sensitive information involved. These datasets do not merely capture a patient’s genetic code but also include detailed data about their current health, lifestyle factors, and environmental exposures. This depth of information increases the risk of privacy breaches and misuse, as it could provide insights that extend far beyond standard genetic data. For instance, a microbiome analysis can reveal dietary habits, geographic origins, or exposure to specific pathogens, while epigenetic data can suggest historical stressors or environmental toxins the patient has encountered. Existing privacy regulations, such as HIPAA and GDPR, may not fully address these complexities, particularly in regard to the ownership, long-term use, and sharing of these data. The challenge of obtaining informed consent becomes more significant, as patients need clear, detailed information about what types of data are being collected, how it will be used, and who will have access to it. Patients may not be aware of how their multi-omics data could be reanalyzed in the future or used for purposes beyond their original consent. To mitigate these risks, advanced encryption and anonymization strategies are essential, but even anonymized data could remain vulnerable to re-identification when combined with other datasets.

Another key ethical challenge involves ensuring equitable access to multi-omics testing. The high costs associated with generating, analyzing, and interpreting multi-omics data may limit its availability to well-resourced healthcare systems or patient populations with comprehensive insurance coverage or significant financial means. Without efforts to democratize access to these advanced diagnostics, there is a risk of exacerbating health disparities, leaving underserved populations without access to personalized medicine innovations. Efforts to develop more cost-effective assays and integrate these tests into public health programs will be crucial in preventing the creation of a “genomic divide.” Regulatory agencies may also need to intervene by mandating insurance providers to cover multi-omics testing when clear clinical benefits are demonstrated.

Finally, there is the challenge of data integration and interpretation. Multi-omics data presents a vast amount of complex information, which can be difficult for clinicians to interpret without advanced clinical decision support (CDS) tools. These tools are vital for processing and translating multi-dimensional data into clear, actionable insights for treatment decisions. However, the development and implementation of robust CDS tools specifically designed to integrate multi-omics data are still in its early stages, leaving clinicians with limited resources to guide decision-making in this emerging field. Moreover, there is a risk of misinterpretation or over-reliance on emerging data without sufficient clinical validation. Multi-omics data are still an evolving field, and, while certain biomarkers or pathways may show promise, they may not yet be fully understood or clinically validated. Without clear guidelines or evidence, clinicians may inadvertently base treatment decisions on unvalidated clinical findings that are not yet sufficiently supported by robust data. Relying on such findings could lead to suboptimal treatment outcomes or even harm. Therefore, ensuring that data interpretation is grounded in validated, evidence-based practices is critical for the safe integration of multi-omics into personalized medicine.

As pharmacogenomics has reached a tipping point in becoming a standard of care, driven by factors such as clinical utility, user acceptance, and economic value [42], the inclusion of related fields like epigenetics, metabolomics, and other multi-omics approaches will further enhance the clinical relevance of personalized medicine. These emerging areas provide deeper insights into drug response and patient outcomes, allowing for even greater precision in personalized healthcare. By incorporating additional layers of biological data—such as epigenetics, metabolomics, and other multi-omics approaches—alongside pharmacogenomics, we can refine therapeutic strategies, enhance drug efficacy, and minimize adverse effects, ultimately accelerating the broader adoption of personalized medicine.

## 9. Conclusions

The integration of epigenetics, nutrigenomics, microbiomes, protein interactions, exosomes, and metabolomics with pharmacogenomics significantly enhances the predictive power of personalized medicine. These approaches promise more precise predictive models, dynamic biomarkers, improved risk stratification, and better prediction of adverse drug events. As we move toward clinical application, addressing the challenges of data utilization and integration becomes crucial. The development of specialized clinical decision support (CDS) tools is essential for clinicians to manage the complexity of multi-omics data and make informed, evidence-based decisions.

Equally important are the ethical and regulatory considerations, including data privacy, patient consent, and equitable access, which must be prioritized to ensure the responsible and fair use of multi-omics in healthcare. By focusing on improvements in medication adherence, appropriateness, and reducing adverse events (MA^3^), we can enhance the precision and effectiveness of personalized treatments. Ultimately, these advancements will improve patient outcomes and quality of life, ensuring that personalized medicine achieves its full potential.

## Data Availability

No new data were created or analyzed in this study. Data sharing is not applicable to this article.
